# Immunological and molecular assessment of HIV-1 mutations for antiretroviral drug resistance in Saudi Arabia

**DOI:** 10.1371/journal.pone.0304408

**Published:** 2024-06-26

**Authors:** Mai M. El-Daly, Kawther A. Zaher, Eitezaz A. Zaki, Leena H. Bajrai, Mohammad M. Alhazmi, Ahmed Abdulhaq, Esam I. Azhar

**Affiliations:** 1 Special Infectious Agents Unit-BSL3, King Fahd Medical Research Center, King Abdulaziz University, Jeddah, Saudi Arabia; 2 Department of Medical Laboratory Sciences, Faculty of Applied Medical Sciences, King Abdulaziz University, Jeddah, Saudi Arabia; 3 Immunology Unit, King Fahd Medical Research Center, King Abdulaziz University, Jeddah, Saudi Arabia; 4 Department of Virology, Jeddah Regional Lab, Ministry of Health, Jeddah, Saudi Arabia; 5 Biochemistry Department, Faculty of Science, King Abdulaziz University, Jeddah, Saudi Arabia; 6 FACP, Arab Board of Internal Medicine, Saudi Board of Internal Medicine, Jazan, Saudi Arabia; 7 Deanship of Scientific Affairs and Research, Jazan University, Jazan, Saudi Arabia; Nigerian Institute of Medical Research, NIGERIA

## Abstract

Human Immunodeficiency Virus (HIV) is a significant threat to public health. HIV genotyping and antiretroviral resistance testing may have contributed to improved non-treated management. Immune markers might assist HIV-1 diagnosis and drug-resistant variant identification. HIV-1 immunogenicity and molecular characteristics of antiretroviral drug resistance are evaluated in 56 treatment-naive HIV patients. DNA sequencing and retroviral resistance testing identified HIV-1 genotypes. 55.4% of patients were susceptible to protease inhibitors (PI), nucleoside reverse transcriptase inhibitors (NRTI), and non-nucleoside reverse transcriptase inhibitors (NNRTI) antiretroviral drugs, whereas 44.6% had drug-resistance mutations against at least one antiretroviral drug. 3.6% of cases had PI-resistant mutations, while 30.4% had NRTI-resistant mutations, and 30.4% had NNRTI-resistant mutations. In patients who are susceptible to PI, the mean value of human plasma sCD80 is 2.11 ± 0.65 ng/mL; in patients with mutations, it is 3.93 ± 2.91 ng/mL. Individuals who are susceptible to PI have plasma sCD27 levels of 78.7 ± 63.2 U/mL, whereas individuals who are mutant have levels of 56.5 ± 32.1 U/mL. IP-10’s mean value was 363 ± 109.2 pg/mL for the susceptible patients and 429 ± 20.7 pg/mL for the mutated patients. In susceptible patients, the plasma sCD4 level is 0.163 ± 0.229 ng/mL; in mutant patients, it is 0.084 ± 0.012 ng/mL. The data showed a relative relation between immunological parameters such as sCD80, sCD27, sCD4, and IP-10 and mutation for drug resistance.

## 1. Introduction

Despite 40 years of progress, the AIDS pandemic is still widespread. Human immunodeficiency virus (HIV) is a Lentivirus-related Retroviridae virus [1]. Two identical single-stranded positive-sense RNAs generate a virus with a diameter between 80 and 120 nm [2]. HIV may manifest in two different forms: HIV-1 and HIV-2 [3]. There are four groups of HIV-1, which are referred to as major (M), outlier (O), neither (non-M/non-O) or new (N), and peripheral (P) [4, 5]. In addition, gene sequences belonging to the M group may be broken down into nine distinct subtypes: *A*, *B*, *C*, *D*, *F*, *G*, *H*, and *K*. Group M is considered a pandemic because it causes pandemics [6].

40.4 million [32.9–51.3 million] people are living with HIV worldwide, 1.3 million people have just been diagnosed with HIV, and 630000 people have passed away due to AIDS in 2022. By 2025, 95% of all persons living with HIV (PLHIV) should receive a diagnosis, 95% should be receiving lifesaving antiretroviral therapy (ART), and 95% of PLHIV on treatment should have a suppressed viral load for the benefit of the person’s health and to reduce forward HIV transmission. These percentages were 86% [73- >98%], 89% [75- >98%], and 93% [79- >98%] in 2022, respectively [7].

The FDA has approved over 25 antiretroviral drugs (ARV) in six classes [8, 9]. Resistance to ARV, which is caused by viral mutations that diminish drug sensitivity, is the primary cause of HIV treatment failure [10, 11]. Molecular tests detect HIV drug-resistance mutations [12]. Antiretroviral therapy (ART) inhibits viral replication and restores immune function in immunocompromised individuals [13]. Antiretroviral therapy aims to reduce the rates of morbidity associated with HIV-1 [14] and death at all stages of illness [15–17], decrease HIV transmission, control plasma viremia [18], and ensure the durable prevention of drug-resistance mutations [19–21].

For HIV-1-infected individuals with no prior therapy, sCD8 levels rise, while sCD4 numbers fall. However, viral load and sCD4 counts were the most significant disease progression indicators. HIV may terminate so many sCD4 cells that the immune system is unable to combat infections without medicine. Untreated HIV infection inhibits the formation, proliferation, cytokine production, and lytic activity of sCD8+ T cells. Untreated HIV viremia causes dysregulation of sCD8+ T cells [23]. Antiretroviral therapy was not reported to recover the loss of resting memory B cells (sCD21 and sCD27 expression) caused by HIV-1 infection. HIV infection is linked to abnormally high plasma concentrations of the proinflammatory chemokine IP-10 [22–25].

Limited studies have reported HIV drug resistance data in Saudi Arabia [26]. Jamjoom et al. conducted a study in Jeddah in 2010 to evaluate HIV-1 reverse transcriptase (RT) and protease (PR) genotyping as well as antiretroviral resistance in HIV-treated individuals. They showed that 41% of the patients had mutations known to cause high-level resistance to one or more nucleoside reverse transcriptase inhibitors (NRTIs), 16% had mutations known to cause high-level resistance to non-nucleoside reverse transcriptase inhibitors (NNRTIs), and 13% had mutations known to cause high-level resistance to protease inhibitors (PIs) [27]. In another study, Al-Mozaini et al. [28] showed that NRTI resistance was the most prevalent (68%), followed by NNRTI resistance mutations (47%) and PI resistance mutations (47%). 77.2% of patients were susceptible to the three major antiretroviral therapy classes: PI, NRTI, and NNRTI. In our recent study [29], we have identified NRTI, NNRTI, and PI resistance mutations in HIV-Naiive patients in Jizan, Southern Saudi Arabia. Our results showed that the prevalence of the mutations was 8.8%, 7.0%, 5.3%, and 1.8% for the NRTI, PI, NNRTI, and both NRTI & PI, respectively.

In this study, we extend our previous report on the resistance-associated mutations in HIV-1 and include immunological parameters reported to be associated with HIV drug resistance. The immunological parameters include levels of sCD4, sCD80, sCD27, and IP-10 and their correlation with drug resistance mutations to select the suitable drug in choice depending on statistical correlation with the immunological parameters.

## 2. Materials and methods

### 2.1 Samples

Blood samples were collected from 56 treatment-naive patients who did not receive any treatment between February 1, 2016, and August 31, 2017, at the HIV clinic at King Fahd Central Hospital in Jazan, Saudi Arabia. The samples were shipped to the Special Infectious Agents Unit, King Fahd Medical Research Center, King Abdulaziz University in Jeddah, Saudi Arabia, for laboratory examination. Ethical approval was obtained from the Biomedical Ethics Unit at King Fahd Central Hospital, Jazan, Saudi Arabia.

### 2.2 Viral serological investigations (EIA)

Plasma samples were evaluated for HIV P24 Antigen and Antibodies with the Genscreen ULTRA HIV Ag-Ab Score kit (BIO-RAD, Germany) following manufacturer instructions.

### 2.3 Isolation of RNA and sequencing

Total RNA was isolated from plasma samples using the Roche MagNA pure compact Nucleic Acid Isolation Kit (Roche, NY, USA). The Abbott Real-Time HIV-1 Assay (Abbott, Germany) was used to evaluate the HIV-1 RNA viral load; Results were analyzed using manufacturer’s guidelines. HIV genotyping was performed for samples with a viral load greater than 1000 Copies/mL, according to Zhou et al., [30], by amplifying the recovered RNA using nested RT-PCR, producing a 1.1 kb fragment of the HIV-1 pol gene region. This fragment included the whole PR region and about two-thirds of the RT region. The One-Step RT-PCR Kit from QIAGEN, Germany, was used to perform RT-PCR on a Veriti 96 well thermal Cycler (Applied BioSystems, Singapore). A fragment of 1084 bp was detected by electrophoresis on an ethidium bromide-stained 1% agarose gel. The nested PCR products were purified using the QIAquick PCR Purification kit (QIAGEN, Germany) according to manufacturer’s instructions. A 3500 DNA genetic instrument was utilized to perform DNA sequencing on purified DNA (Applied Biosystems, USA). The entire region was sequenced using three pairs of primers. The raw sequencing results were assembled using Geneious 8.1.5 software (Biomatters, Auckland, New Zealand). To determine the HIV-1 genotypes, the obtained sequences were aligned using ClustalW with HIV reference sequences from the National Center for Biotechnology Information (NCBI) library (https://blast.ncbi.nlm.nih.gov/Blast).

### 2.4 Identification of HIV-1 genotypes and drug resistance

The Stanford Genotypic Resistance Interpretation Algorithm was used to assess HIV-1 resistance-associated mutations in the PR and RT regions (http://hivdb.stanford.edu/pages/algs/HIVdb.html).

### 2.5 Immunological evaluation

#### 2.5.1 Plasma soluble sCD80 level detection

For the quantitative detection of human sCD80, an enzyme-linked immunosorbent assay (ELISA) was performed using an Invitrogen® kit (Thermo Fisher Scientific, Catalog# BMS291INST), and the procedure was carried out following the manufacturer instructions.

#### 2.5.2 Plasma soluble sCD27 level detection

The Human sCD27 Immediate ELISA Kit, Invitrogen® (Thermo Fisher Scientific, Bender MedSystems GmbH, Vienna, Aus.; Catalog# BMS286INST) was used to assess plasma sCD27 levels according to manufacturer instructions.

#### 2.5.3 Detection of plasma levels of IP-10

The Human IP-10 ELISA Kit Invitrogen® (Invitrogen Company, CA 930, USA; Catalog# KAC2361) was used to evaluate IP-10 levels in cases recruited in this study according to manufacturer instructions.

#### 2.5.4 Measurement of sCD4 concentrations in plasma

The Human sCD4 Simple-Step ELISA® Kit determined the soluble sCD4 plasma concentration (Abcam, Japan, Catalog# ab234569 according to manufacturer instructions.

### 2.6 Statistical assessment of data

The data were analyzed using the Statistical Program for the Social Sciences (SPSS version 21, IBM, New York, United States), with categorical data reported as frequency and percentage (%) and continuous data supplied as mean and SE. The analysis of immunological data was performed using the Kruskal-Wallis test. Continuous variables were analyzed using the ANOVA test; categorical variables were compared using the Chi-square test.

## 3. Results

### 3.1 Patients’ general characteristics

A total of fifty-six non-treated HIV-1 patients were recruited from King Fahd General Hospital in Jazan (South-Western Saudi Arabia), of whom 37 (66.1%) were Saudi, and 19 (33.9%) were non-Saudi; 36 (64.3%) were male and 20 (35.7%) were female, and their mean age was 35.0 years.

### 3.2 Molecular assessments

#### 3.2.1 Prevalence of genotypes and their relationship to mutations

The sequencing analysis showed that 50% (28/56) of individuals had genotype *C*. Genotype *G* was the second highest frequency at 14.3% (8/56), followed by genotypes *B* and *D* at 7.1% (4/56) each, genotype *A* at 5.4% (3/56), and genotypes *CRF02 AG*, *CRF01_AE*, and *G+CRF02_AG* at 3.6% (2/56) each. The frequency of each genotype *CRF09 cpx*, *CRF16 A2D*, and *CRF45 cpx* was 1.8% (1/56).

**Fig 1:**
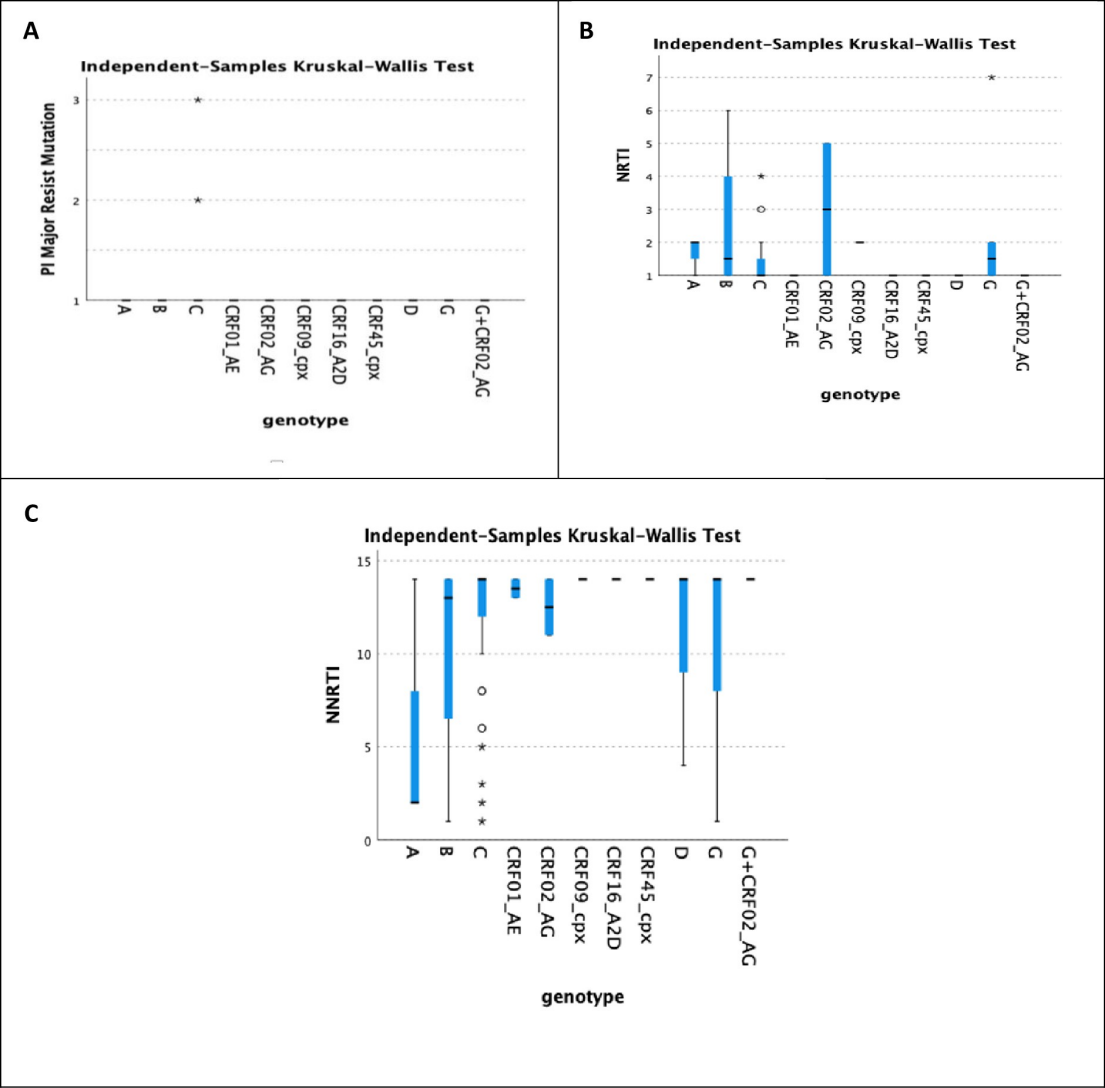
The relation between resistance mutations and genotypes. A indicates a correlation between PI major resistance mutations and genotype C. B indicates that NRTI resistance mutations are mainly associated with genotypes A, B, C, G, and CRF02_AC. C illustrates a correlation between NNRTI resistance mutations and genotypes A, B, C, CRF01_AE, CRF02_AC, D, and G.

PI Major resistance mutations were detected only in genotype *C* (Fig 1A). In contrast, NRTI resistance mutations were detected in genotypes *A*, *B*, *C*, *CRF02 AG*, *CRF09 CPX*, and *G* (Fig 1B), and NNRTI resistance mutations were detected in genotypes in all detected genotypes (Fig 1C). There are no significant correlations between PI major resistance (*p* = 0.996), NRTI resistance (*p* = 0.376), NNRTI resistance (*p* = 0.8), and HIV-1 genotypes.

#### 3.2.2 Mutational resistance to antiretroviral drugs

**Fig 2:**
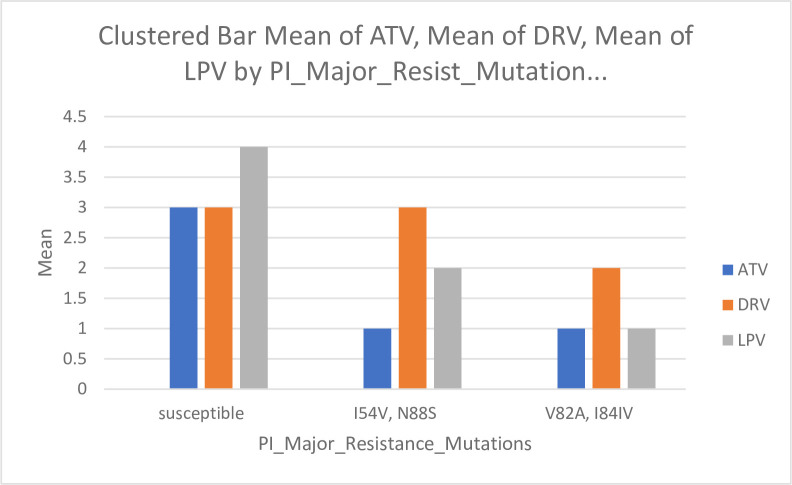
PI major resistance mutations to different drugs. Fig 2 displays the relationship between the susceptibility and mutations of PI Major Resistance Mutations to three different drugs: atazanavir (ATV), darunavir (DRV), and lopinavir (LPV). The left columns show susceptibility to the drugs ATV, DRV, and LPV. The middle columns show mutations (I54V and N88S), and the right columns show mutations (V82A and I84IV).

*3*.*2*.*2*.*1*. *PI major resistance mutations to different drugs*. The mutations (I54V, N88S), and (V82A, I84IV) were reported to be significantly associated with high-level resistance to the drug atazanavir (ATV) and were detected in 1.8% of the recruited cases each (*p*<0.001), and 96.4% of the cases were susceptible to ATV. For the darunavir (DRV) drug, the mutations (I54V, N88S) were reported to infer susceptibility to the drug, while the mutations (V82A, I84IV) were reported to be significantly associated with low-level resistance to the drug in 1.8% of the recruited cases (*p*<0.001), and 96.4% of the cases were susceptible to DRV. Mutations (V82A, I84IV) reported to be significantly associated with high-level resistance to lopinavir (LPV), and mutations (I54V, N88S) were reported to be significantly associated with low-level resistance to LPV were detected in 1.8% of the recruited cases each (*p*<0.001), and 96.4% of the cases were susceptible to LPV (Fig 2).

**Fig 3:**
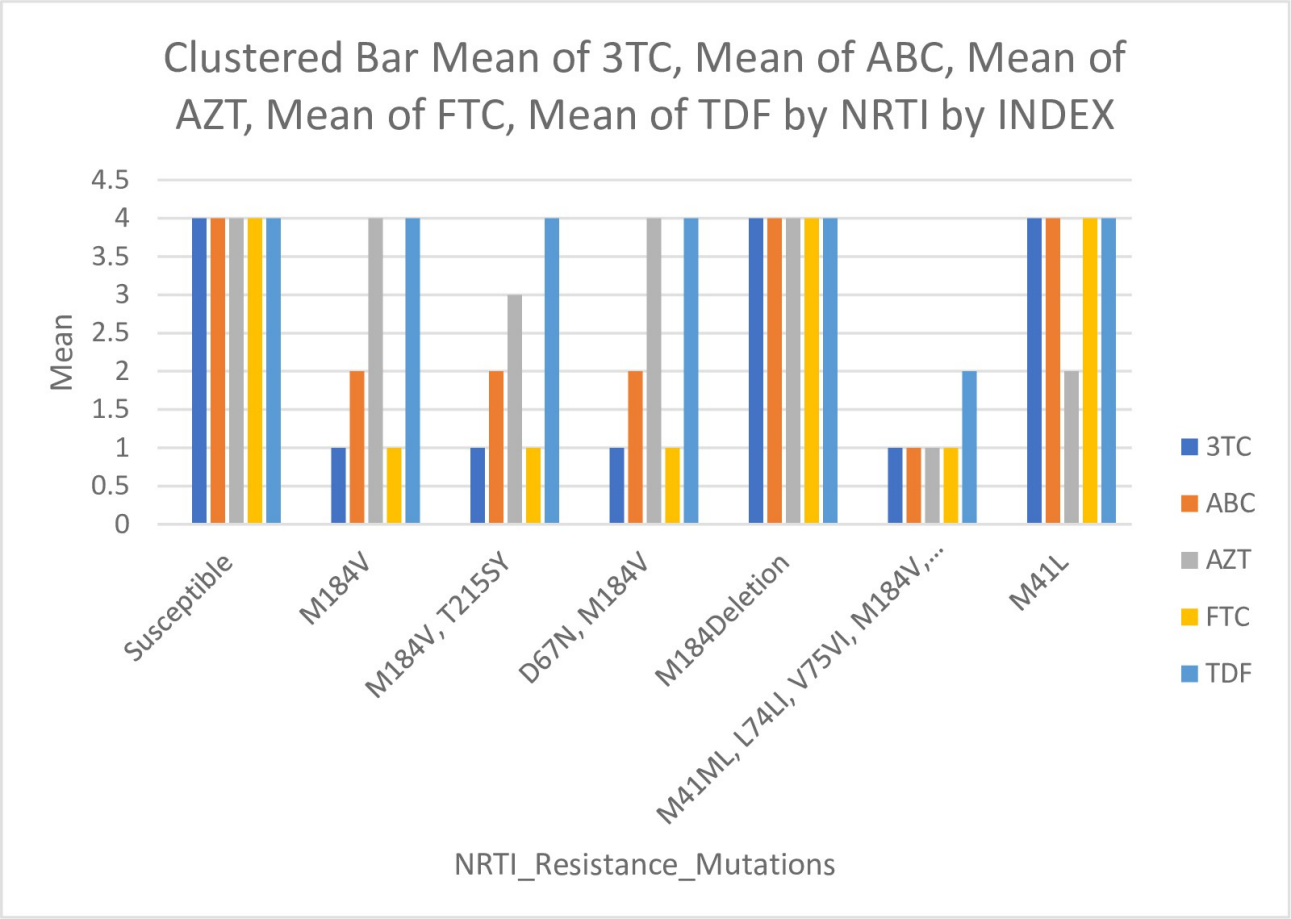
Correlation of NRTI resistance mutations with different drugs. It was found that 17 patients had 9 different NRTI resistance mutations to the drugs such as lamivudine (3TC), abacavir (ABC), zidovudine (AZT), emtricitabine (FTC), and tenofovir (TDF). Among these patients, the M184V mutation was detected as a single mutation in 12 patients and a combination with other mutations in 3 patients.

*3*.*2*.*2*.*2 NRTI resistance mutations to different drugs*. The M184V mutations, associated with high-level resistance to lamivudine (3TC) and emtricitabine (FTC), were observed in 21.4% of patients. The mutations (D67N, M184V), (M184V, T215SY), and (M41ML, L74LI, V75VI, M184V, T215TNS) were highly significant associated with high-level resistance, represented by 1.8% each (*p*<0.001). M184 Deletion and M41L showed highly significant susceptibility to lamivudine (3TC), represented by 1.8% each (*p*<0.001), and 69.6% of the patients were susceptible to the drug (Fig 3).

The mutations (D67N, M184V), (M184V, T215SY), and (M41ML, L74LI, V75VI, M184V, T215TNS) were highly significantly associated with high-level resistance to emtricitabine (FTC), represented by 1.8% each (*p*<0.001). M184 Deletion and M41L showed highly significant susceptibility to FTC, represented by 1.8% each (*p*<0.001), and 69.6% of the patients were susceptible to FTC (Fig 3).

For the abacavir (ABC) drug, mutations (M41ML, L74LI, V75VI, M184V, T215TNS) were highly significant associated with high-level resistance to the drug, represented by 1.8% (*p*<0.001). The mutations M184V, (D67N, M184V), and (M184V, T215SY) were highly significant associated with low-level resistance to the drug, represented by 21.4%, 1.8%, and 1.8% respectively (*p*<0.001). M184 Deletion and M41L showed highly significant susceptibility, represented by 1.8% each (*p*<0.001), and 69.6% of the patients were susceptible to the drug (Fig 3).

For the zidovudine (AZT) drug, mutations (M41ML, L74LI, V75VI, M184V, T215TNS) were highly significant associated with high-level resistance to the drug, represented by 1.8% (*p*<0.001). Mutations (M184V, T215SY) were highly significantly associated with intermediate resistance to the drug, represented by 1.8% (*p*<0.001). Mutation M41L was highly significantly associated with low-level resistance to the drug, represented by 1.8% (*p*<0.001). Mutations M184V, (D67N, M184V), and M184 Deletion showed highly significant susceptibility represented by 21.4%, 1.8%, and 1.8%, respectively (*p*<0.001), and 69.6% of the patients were susceptible to the drug (Fig 3).

For the tenofovir (TDF) drug, mutations (M41ML, L74LI, V75VI, M184V, T215TNS) were highly significant associated with low-level resistance to the drug, represented by 1.8% (*p*<0.001). Mutations (D67N, M184V), M184Deletion, (M184V, T215SY), and M41L showed highly significant susceptibility represented by 1.8% each, while M184V represented by 21.4% (*p*<0.001), and 69.6% of the patients were susceptible to the drug (Fig 3).

**Fig 4:**
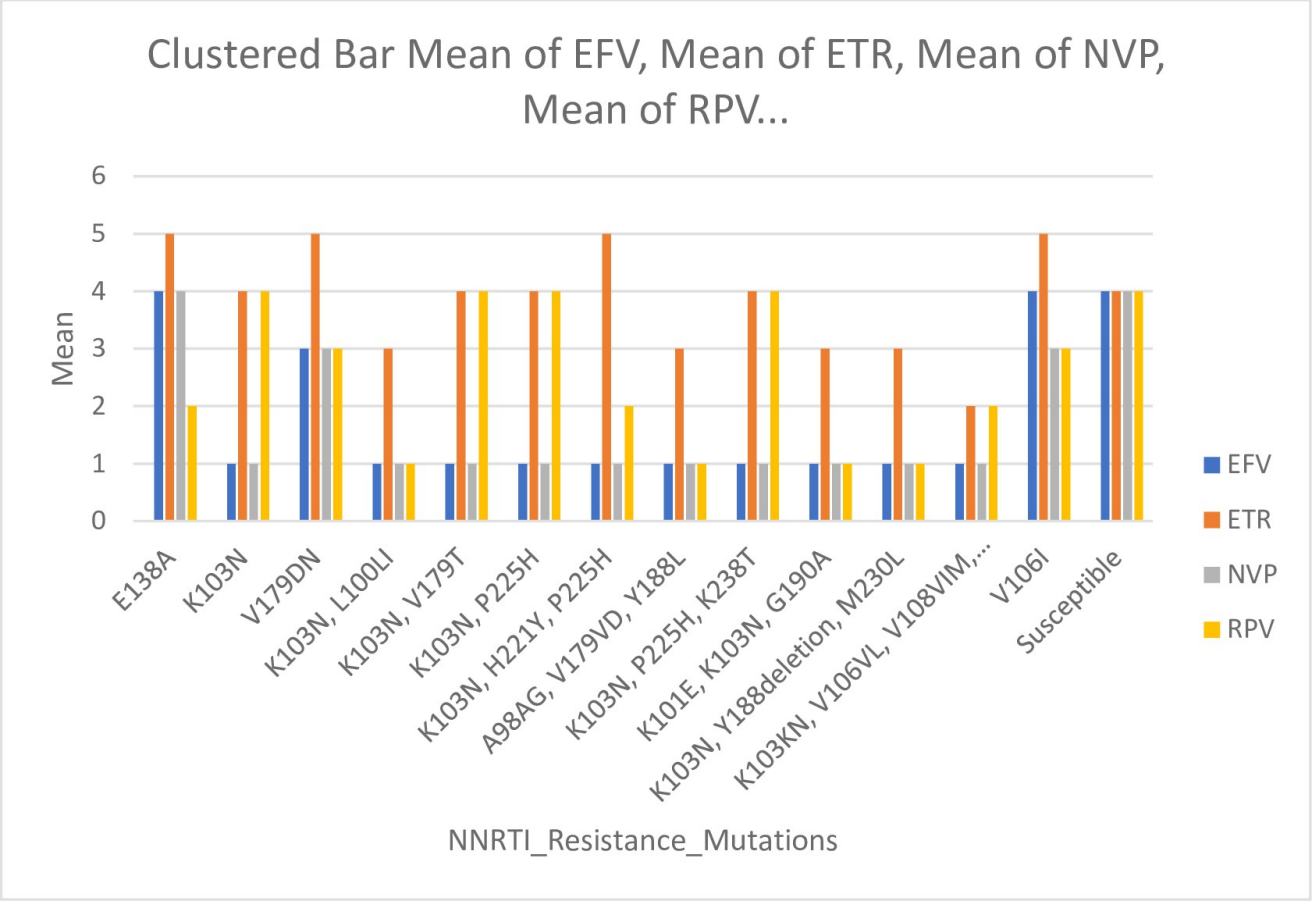
Correlation of NNRTI resistance mutations with different drugs. Fig 4 displays the susceptibility and mutations of NNRTI Resistance for four different drugs: efavirenz (EFV), etravirine (ETR), nevirapine (NVP), and rilpivirine (RPV). The figure includes data from 17 patients with various mutations, while the right columns indicate the drugs’ susceptibility towards these mutations.

*3*.*2*.*2*.*3*. *NNRTI resistance mutations to different drugs*. For the efavirenz (EFV) drug, mutation K103N was highly significantly associated with high-level resistance to the drug, represented by 5.4%. In contrast, the mutations (K103N, L100LI), (K103N, V179T), (K103N, P225H), (K103N, H221Y, P225H), (A98AG, V179VD, Y188L), (K103N, P225H, K238T), (K101E, K103N, G190A), (K103N, Y188Del, M230L), and (K103KN, V106VI, V108VIM, E138EA) were highly significant associated with high-level resistance to the drug, represented by 1.8% each (*p*<0.001). The V179DV mutation was highly significantly related to potential low-level resistance to the drug, characterized by 1.8% (*p*<0.001). Mutations E138A and V106I were highly significant susceptibility, represented by 1.8% each (*p*<0.001), and 69.6% of the patients were susceptible to the drug (Fig 4).

For the etravirine (ETR) drug, mutations (K103N, L100LI), (A98AG, V179VD, Y188L), (K101E, K103N, G190A), and (K103N, Y188Del, M230L) were highly significant associated with intermediate resistance to the drug, represented by 1.8% each (*p*<0.001). Mutations (K103KN, V106VI, V108VIM, E138EA) were highly significantly associated with low-level resistance to the drug, represented by 1.8% (*p*<0.001). Mutations V179DV, (K103N, H221Y, P225H), and V106I were highly associated with potential low-level resistance to the drug, represented by 1.8% each, while the E138A mutation was described by 5.4% (*p*<0.001). Mutations (K103N, V179T), (K103N, P225H), and (K103N, P225H, K238T) were highly significant susceptibility represented by 1.8% each, while K103N mutation described by 5.4% (*p*<0.001), and 69.6% of the patients were susceptible to the drug (Fig 4).

For the nevirapine (NVP) drug, mutations (K103N, L100LI), (K103N, V179T), (K103N, P225H), (K103N, H221Y, P225H), (A98AG, V179VD, Y188L), (K103N, P225H, K238T), (K101E, K103N, G190A), (K103N, Y188Del, M230L), and (K103KN, V106VI, V108VIM, E138EA) were highly significant associated with high-level resistance to the drug, represented by 1.8% each, while mutation K103N represented by 5.4% (*p*<0.001). The V179DV and V106I mutations were highly significant associated with potential low-level resistance to the drug, represented by 1.8% each (*p*<0.001). Mutation E138A was highly significant susceptibility, represented by 5.4% (*p*<0.001), and 69.6% of the patients were susceptible to the drug (Fig 4).

For the rilpivirine (RPV) drug, mutations (K103N, L100LI), (A98AG, V179VD, Y188L), (K101E, K103N, G190A), and (K103N, Y188Del, M230L) were highly significant associated with high-level resistance to the drug, represented by 1.8% each (*p*<0.001). Mutations (K103N, H221Y, P225H) and (K103KN, V106VI, V108VIM, and E138EA) were highly associated with low-level resistance to the drug, represented by 1.8% each, while the E138A mutation was represented by 5.4% (*p*<0.001). Mutations V179DV and V106I were highly associated with potential low-level resistance to the drug, represented by 1.8% each (*p*<0.001). Mutations (K103N, V179T), (K103N, P225H), (K103N, P225H, K238T), and were highly significant susceptibility represented by 1.8% each, while K103N mutation described by 5.4% (*p*<0.001), and 69.6% of the patients were susceptible to the drug (Fig 4).

### 3.3 Immunological assessment

#### 3.3.1 Detection of soluble sCD80 in plasma

Comparing data of susceptible and mutant patients, the mean values of sCD80 in plasma were 2.11 ± 0.65 ng/mL and 2.24 ± 1.01 ng/mL, respectively. The minimum values were 0.65 ng/mL and 1.01 ng/mL, respectively, while the maximums were 3.32 ng/mL and 5.99 ng/mL, respectively, with the *p*-value 0.28.

#### 3.3.2 Detection of soluble sCD27 in plasma

The data for susceptible and mutant patients showed that the mean values were 73.6 ± 46.4 U/mL and 83.2 ± 78.4 U/mL, respectively. The maximum values were 244.6 U/mL and 377.4 U/mL. On the other hand, the minimum values were 12.6 U/mL and 13.0 U/mL, respectively, and the *p*-value was 0.27.

#### 3.3.3 Detection of IP-10 levels in plasma

The levels of IP-10 in the plasma of susceptible and mutant patients showed a mean value of 382 ±86.9 pg/mL and 344 ±128 pg/mL; the minimum values were 86.9 pg/mL and 26 pg/mL, and maximum values were 484 pg/mL and 485 pg/mL, respectively, with a *p*-value 0.07.

#### 3.3.4 Detection of soluble sCD4 in plasma

Comparing data of susceptible and mutant patients, the mean values of sCD4 were 0.177 ± 0.179 ng/mL and 0.139 ± 0.274 ng/mL, while the minimums were 0.041 ng/mL and 0.000, and maximum values were 0.902 ng/mL and 1.315 ng/mL, respectively with a *p*-value of 0.25.

### 3.4 Correlation between immunological markers and virus genotype

**Fig 5:**
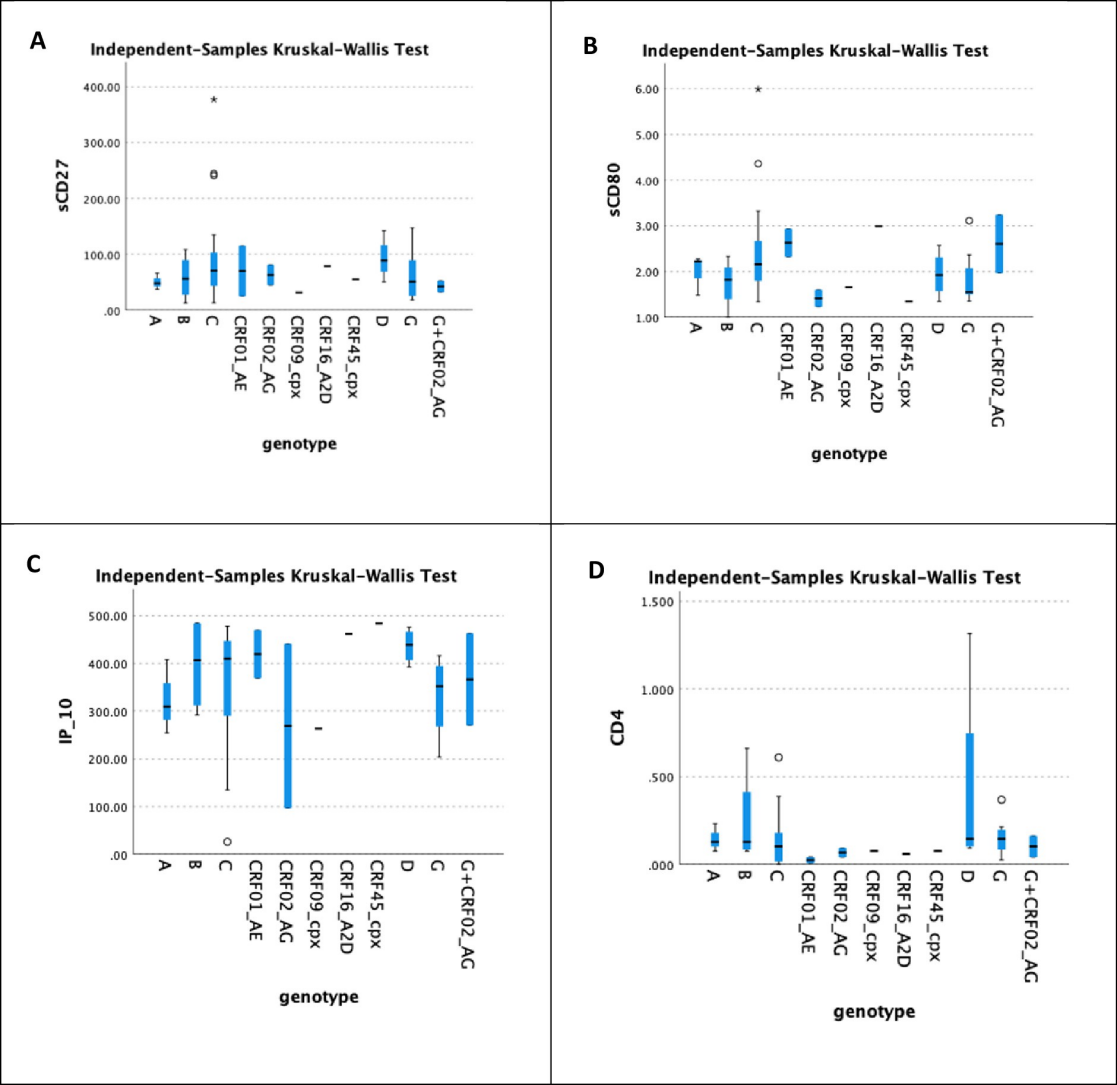
Correlation between different immunological markers and genotypes. A displays the correlation between sCD27 and different genotypes. Fig 5B shows the correlation between sCD80 and genotypes. Fig 5C illustrates the correlation between IP-10 and genotypes. Lastly, Fig 5D portrays the correlation between sCD4 and genotypes.

Fig 5 depicts the distribution of IP-10, sCD27, sCD80, and sCD4 levels across several viral genotypes. Unfortunately, the findings support the null hypothesis, and there is no significant link since the distribution of viral genotypes was the same across categories, yielding *p*-values of 0.306, 0.749, 0.105, and 0.623 for IP-10, sCD27, sCD80, and sCD4 when *p*<0.05.

### 3.5 Correlations between immunological markers and resistant mutations

**Fig 6:**
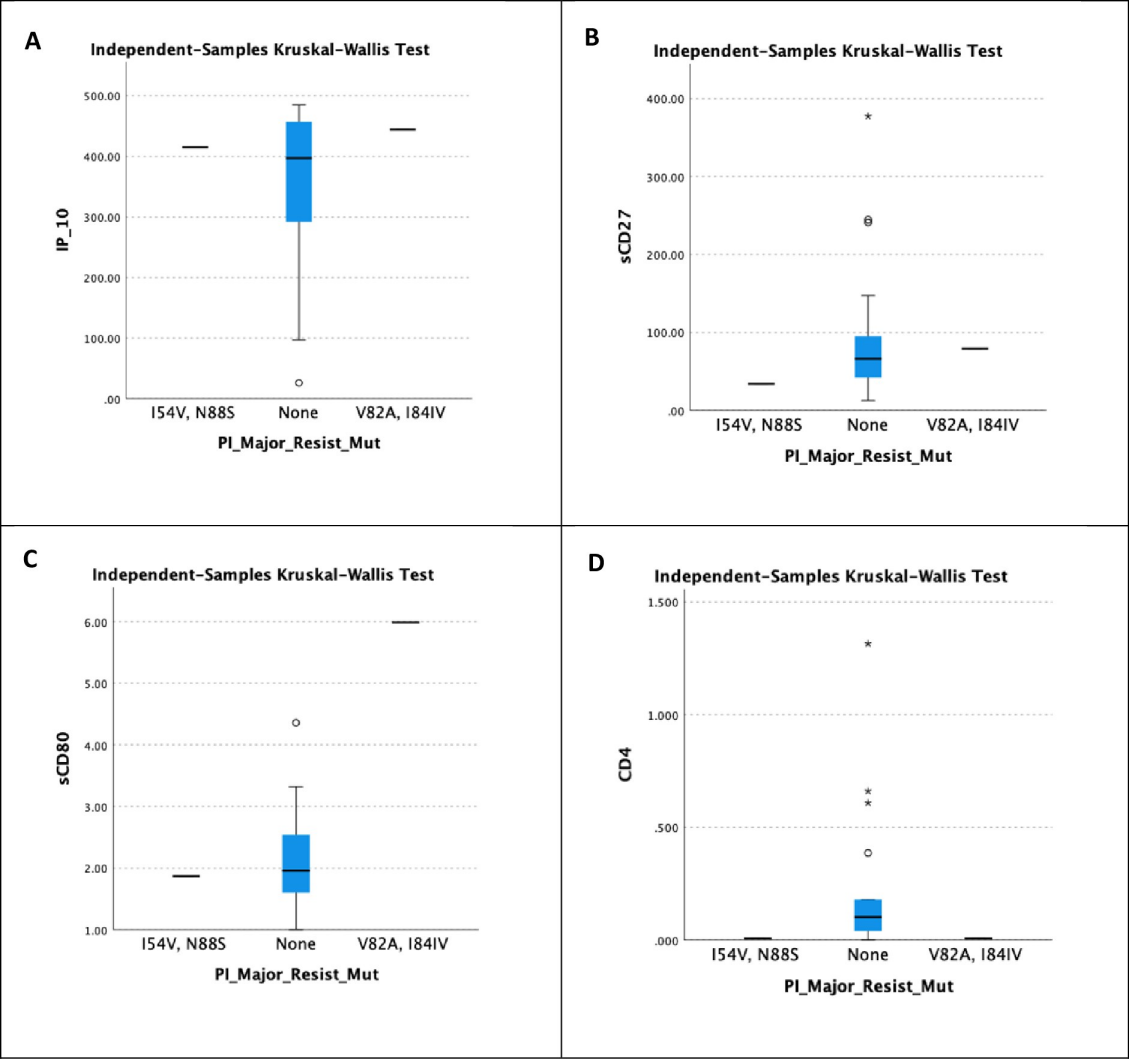
Correlation between different immunological markers and PI major resistance mutations. A demonstrates the correlation between IP-10 and PI major resistant mutation. B illustrates the correlation between sCD27 and PI major resistant mutation. C displays the correlation between sCD80 and PI major resistant mutation. Finally, D shows the correlation between sCD4 and PI major resistant mutation.

Fig 6 depicts the relationship between IP-10, sCD27, sCD80, and sCD4, as well as the major resistance mutation of PI. There were no significant findings for IP-10, sCD27, sCD80, and sCD4, with *p*-values of 0.744, 0.517, 0.231, and 0.163 with *p*-value <0.05.

**Fig 7:**
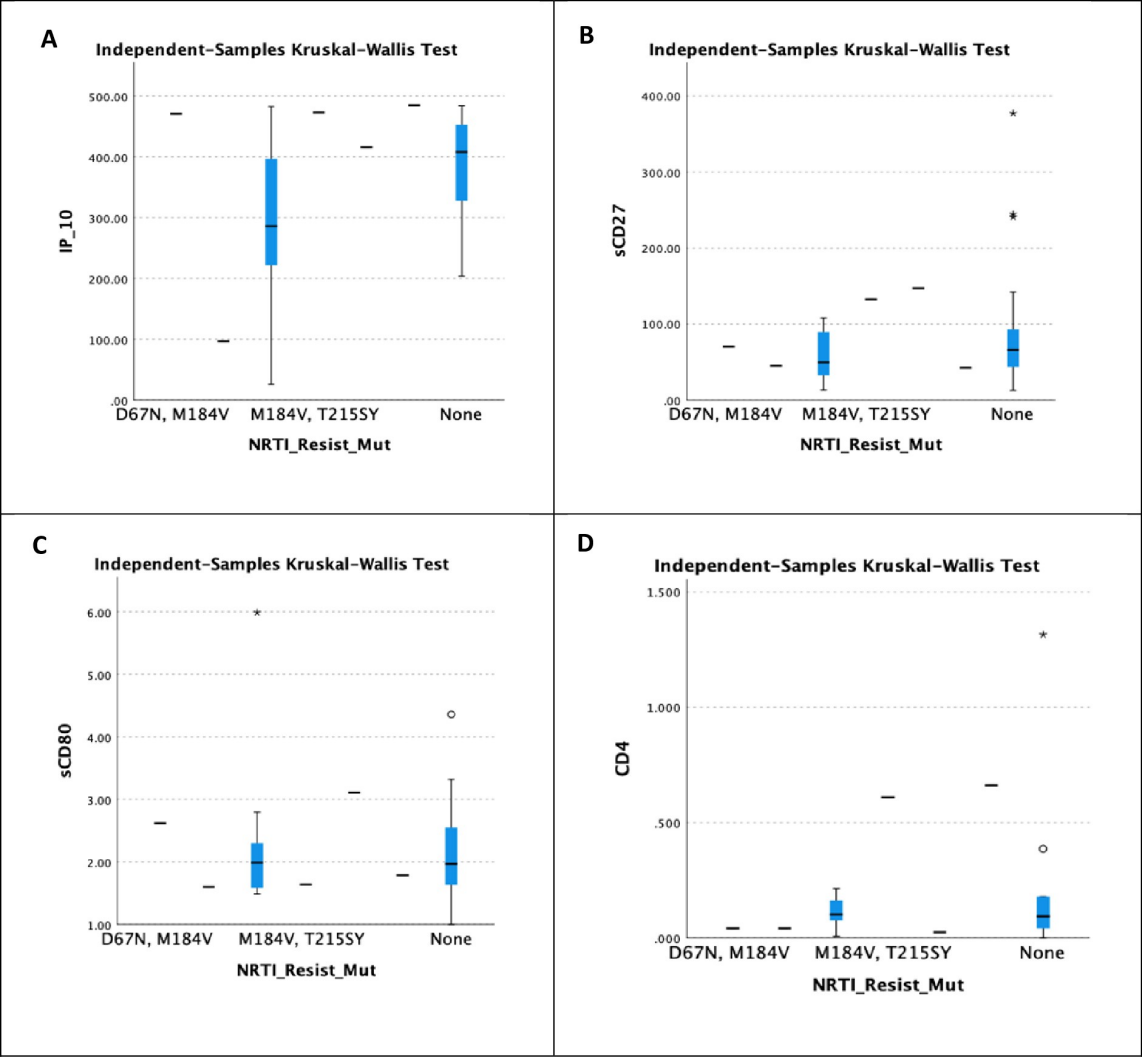
Correlation between different immunological markers and NRTI resistance mutation. A indicates the correlation between IP-10 and NRTI resistant mutation. B shows the correlation between sCD27 and NRTI resistant mutation. C displays the correlation between sCD80 and NRTI resistant mutation. Finally, D portrays the correlation between sCD4 and NRTI resistant mutation.

The distribution of IP-10, sCD27, sCD80, and sCD4 levels across NRTI-resistant mutations was demonstrated in Fig 7. There was a significant association between IP-10 and NRTI resistant mutations with a *p*-value of 0.027. Unfortunately, the findings support the null hypothesis, and there is no significant link since the distribution of NRTI-resistant mutations was the same across categories, yielding values of 0.443, 0.593, and 0.224 for sCD27, sCD80, and sCD4 when *p*-value <0.05.

**Fig 8:**
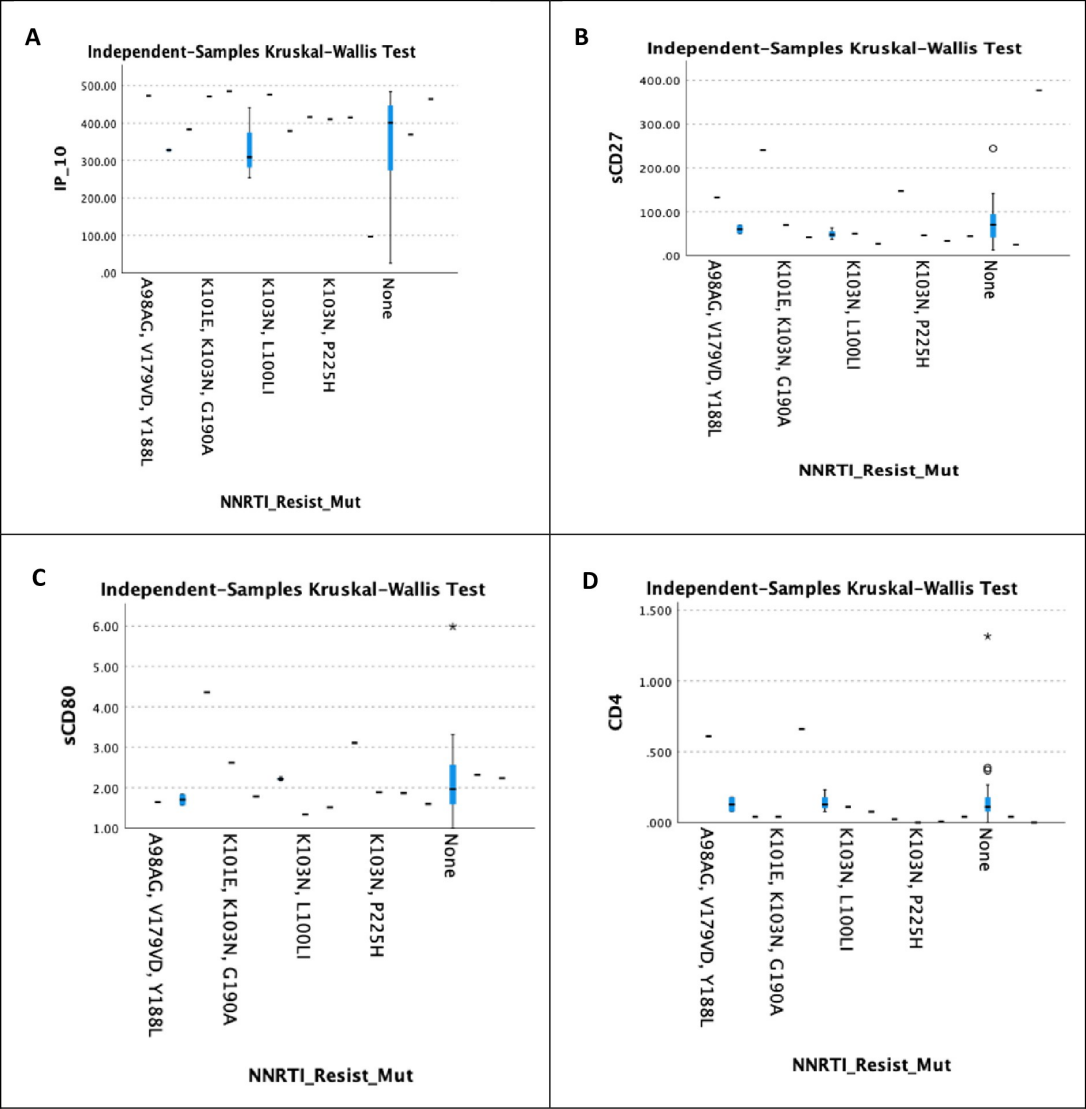
Correlation between different immunological markers and NNRTI resistance mutations. A shows the correlation between IP-10 and NNRTI resistant mutations. B displays the correlation between sCD27 and NNRTI resistant mutations. C indicates the correlation between sCD80 and NNRTI resistant mutations. D demonstrates the correlation between sCD4 and NNRTI resistant mutations.

Fig 8 depicts the relationship between IP-10, sCD27, sCD80, and sCD4 and the NNRTI resistant mutations. There were no significant findings for IP-10, sCD27, sCD80, and sCD4, with respective *p*-values of 0.405, 0.277, 0.589, and 0.208 when *p*<0.05.

## 4. Discussion

HIV drug resistance is caused by changes in HIV genome structure, which reduces the effectiveness of antiviral treatments in restricting virus replication. Since drug-resistant viruses have become more common, all antiretroviral drugs, particularly those of newer drug classes, could eventually lose some or all their effectiveness. Therefore, it is possible that HIV drug resistance could undermine the effectiveness of antiretroviral drugs in their mission to reduce the incidence of HIV as well as morbidity and mortality [19, 31].

In this study, samples were collected from 56 chronic non-treated HIV patients from Jazan, Saudi Arabia, 66.1% of the recruited subjects were Saudis, 33.9% were non-Saudi, 64.3% were male, and 35.7% were female with a mean age of 35.0 years. Sequence analysis showed that genotype *C* was the most frequent genotype with a prevalence of 50.0%, consistent with our earlier findings in which the prevalence was 66.6% [29, 32, 33] while Jamjoom et al. found it was 34% [27]. In contrast, genotype *G* was the second most prevalent genotype at 14.3%, followed by genotypes *B* and *D* at 7.1% each, genotype *A* at 5.4%, and genotypes *CRF02 AG*, *CRF01_AE* and *G+CRF02_AG* 3.6% each. However, the frequency of each genotype *CRF09 cpx*, *CRF16 A2D*, and *CRF45 cpx* was 1.8%. The results are consistent with recent research findings, genotype *C* is widespread in the Horn of Africa, Djibouti, China, and India [34]. Genotype *G* is predominant throughout Europe, Australia, the Americas, some regions of Asia, and the Middle East. Although genotype *B* is most common in the Americas, it is also prevalent in the Middle East, North Africa, Australia, South Asia, and central Europe [34]. Genotype *A* is also found in Europe and Africa [36]. In contrast, the *CRF01 AE* genotype is prevalent in Asia, while the *CRF02 AG* genotype is prevalent in West Africa [35].

The immune profile is a reliable tool for assessing how the body reacts to ARV drugs and how effective the treatment was or was not. sCD8+ T cells are essential to the adaptive immune response generated against infection. It is influenced by the fact that the virus continues to replicate. sCD8 cells and their subpopulation cells are responsible for the major function of secreting cytokines, including IFN, TNF-α, and IL-2. These cytokines are what cause inflammatory responses and have antiviral effects. A significant number of sCD8+ T cells indicates a favorable response to ARV treatment [36–38]. Recent research indicates an increased level of sCD80 in the plasma samples of patients susceptible to ART-listed drugs. sCD80 should not be present in the blood of a person not infected with HIV. Therefore, it is possible that the elevated levels of sCD80 in the blood of HIV-treated patients are connected to the success of the medication, and the data presented here are congruent with research conducted in the past [36, 37]. The result of this study showed high levels of sCD80 (5.99 ng/mL**)** in mutant patients which is higher than in susceptible patients (3.32 ng/mL). For each mutation, the plasma level of sCD80 for PI-susceptible patients was 2.11 ± 0.65 ng/mL, while in mutated patients it was 3.93 ± 2.91 ng/mL. While susceptible patients to NRTI is 2.13 ± 0.70 ng/mL, and in mutated patients are 2.26 ± 1.08 ng/mL. The mean value of sCD80 for the susceptible patients to NNRTI is 2.18 ± 0.87 ng/mL, while in the mutated patients is 2.14 ± 0.72 ng/mL. The levels of sCD80 in mutant patients are higher than in drug-susceptible patients, which comes in agreement with previous studies that sCD80 must be high as there is no treatment for HIV with any of the desired drugs.

The amount of sCD4 differs according to the stage of HIV disease as it decreased when the disease progresses to stages 2 and 3 [22]. In our study, the plasma sCD4 level is 0.163 ± 0.229 ng/mL for the susceptible patients to PI, while in mutated patients it is 0.084 ± 0.012 ng/mL. The sCD4 level for the susceptible patients to NRTI is 0.187 ± 0.250 ng/mL, while in the mutated patients, it is 0.099 ± 0.143 ng/mL. Also, for susceptible patients to NNRTI, the level was 0.171 ± 0.180 ng/mL, and in mutated patients was 0.136 ± 0.310 ng/mL. These results indicate the good prognosis of treatment, which comes in agreement with Warren et al [22].

When sCD4^+^ T cells are activated by an antigen, especially a viral infection, a large amount of sCD27 is generated. This is because sCD27 is an antiviral protein. As a result, many authorities believe that high levels of sCD27 suggest the development of sCD4^+^ T cells that have memory [39]. The levels of sCD27 in human peripheral blood ranged from 70.8 U/mL to 353.5 U/mL, with a mean value of 196.0 U/mL. The levels of sCD27 in the plasma of patients sensitive to ART-listed drugs are greater than those of highly resistant patients. Individuals with a robust immune response to infection have higher levels of sCD27. In the current study, the plasma sCD27 level for the susceptible patients to PI is 78.7 ± 63.2 U/mL; in the mutated patients, it is 56.5 ± 32.1 U/mL. The plasma level for the susceptible patients to NRTI is 82.3 ± 70.0 U/mL, while in the mutated patients is 67.8 ± 39.3 U/mL. While the plasma levels of sCD27 of susceptible patients to NNRTI is 73.1 ± 44.0 U/mL, while in the mutated patients is 88.8 ± 92.5 U/mL which comes in agreement with [39, 41]. sCD27^+^ B is believed to be a memory marker for B cells because of its association with giant, cytoplasm-rich cells known as plasma cells. B cells also can undergo somatic hypermutation and produce immunoglobulins. The binding of sCD27 generates necessary signals that positively govern the entry of B cells into the pathway, ultimately resulting in plasma cells.

IFN-gamma-inducible protein 10, usually called CXCL10, is another name for IP-10. When HIV is present, it promotes the growth of new blood vessels (angiogenesis) and enhances the chemotactic activity of CXR3+ cells, ultimately leading to the death of infected cells. Its level rises after infection with HIV, which has been shown to impede the function of immune cells. Plasma IP-10 values considered normal ranged from 60 to 282 pg/mL. IP-10’s mean value was 363 ± 109.2 pg/mL for the susceptible patients to PI and 429 ± 20.7 pg/mL for the mutated patients. The IP-10 mean value for NRTI-susceptible patients was 386 ± 81.4 pg/mL, and 318 ± 144.6 pg/mL for mutated patients. The mean value of IP-10 was 358 ± 111.9 pg/mL for NNRTI-susceptible patients and 382 ± 99.4 pg/mL for mutated patients. The current samples exhibit significant levels of IP-10, which, following the findings of other studies, are accompanied by high levels of viral load [40].

Some of the research results presented do not show any significancy between the Immune system or resistance to the pharmaceutical profile (Figs 3–5). Despite this, the levels of sCD80, sCD27, and IP-10 indicated the immunological condition of the patients, which is in line with the conclusions that Hattab et al. and Shiau et al. came to [41, 42].

## 5. Conclusions

To address HIV drug resistance, genotyping, and immune profiling should be implemented to determine if HIV treatment is effective, and in cases of proven treatment failure, treatment regimens should be changed rapidly. This research revealed that the predominant HIV-1 genotype in Saudi Arabia is C (50.0%), and the most frequent medication resistances are V82A and I84IV. The mutation was linked to LPV, FTC, ATV, 3TC, ABC, and DRV resistance. The immunological profile of HIV patients indicates the immune response and anti-HIV drug resistance. High levels of sCD80 in mutant patients are higher than in drug-susceptible patients, while the opposite happens in sCD4, IP-10, and sCD27, which reflect the pattern of drug resistance and prognosis of treatment as well. Using immunological profiling can help predict drug resistance before performing genetic mutations through sequencing. Thus, assisting the physician in determining the most effective HIV treatment regimen. As in other clinical settings, HIV infection is linked to increased immune activation in Saudi patients. Many of them are also associated with short-term mortality. Further research is required to determine whether these biomarkers may indicate bad long-term outcomes.

## Supporting information

S1 Checklist Human participants research checklist. (DOCX)

S1 Table (TIF)

S1 File (DOCX)
