# Supplementary figures and images for: Immunological and molecular assessment of HIV-1 mutations for antiretroviral drug resistance in Saudi Arabia
Source: PLoS One. 2024 Jun 26;19(6):e0304408. doi: 10.1371/journal.pone.0304408 (PMC11207162; doi:10.1371/journal.pone.0304408)

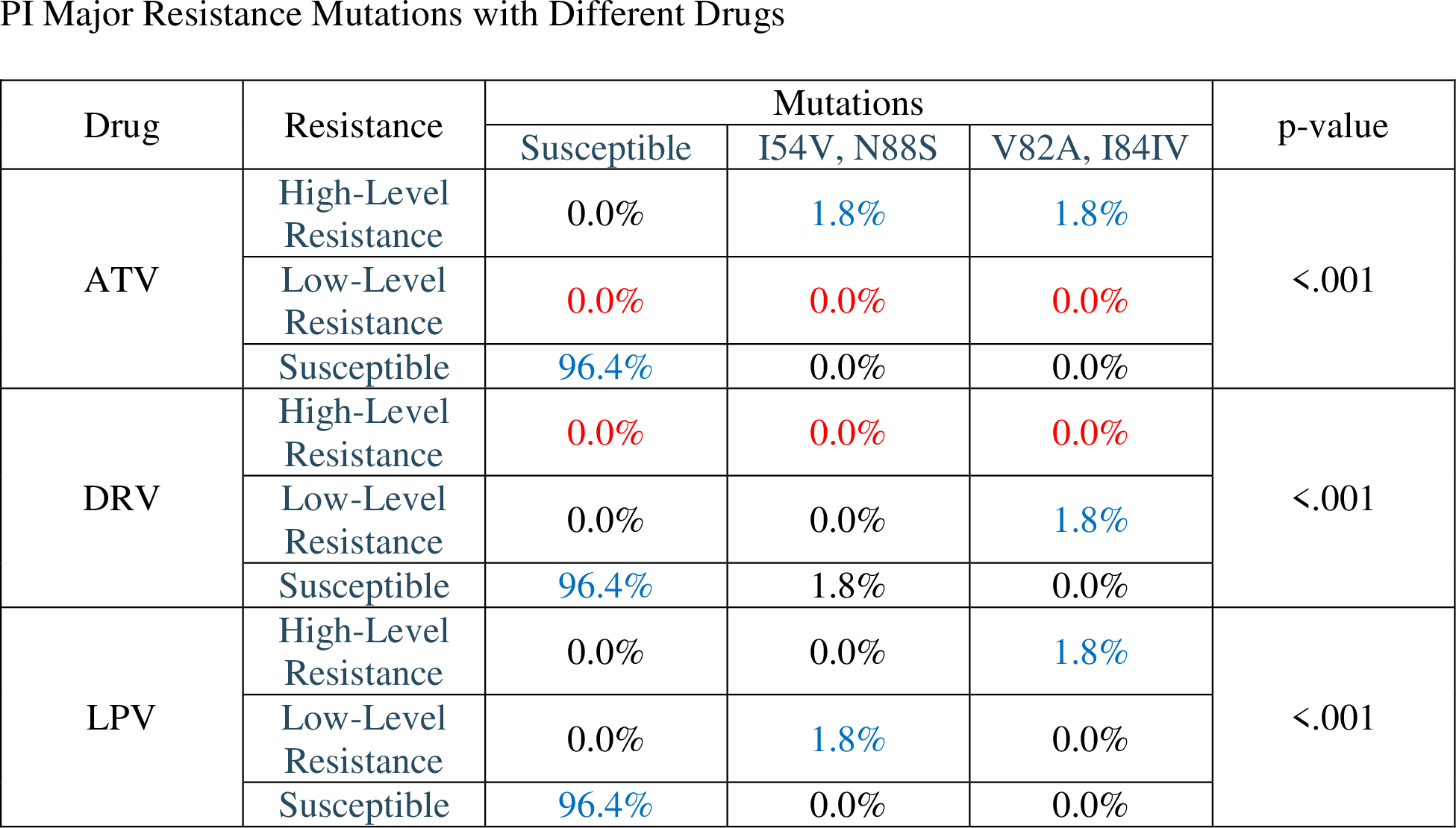

Supplement: S1 Table — (TIF) [file pone.0304408.s002.tif]
