# Supplementary material for: Immunological and molecular assessment of HIV-1 mutations for antiretroviral drug resistance in Saudi Arabia
Source: PLoS One. 2024 Jun 26;19(6):e0304408. doi: 10.1371/journal.pone.0304408 (PMC11207162; doi:10.1371/journal.pone.0304408)
Supplement: S1 File — (DOCX) [file pone.0304408.s003.docx]

PI Major Resistance Mutations with Different Drugs

| Drug | Resistance | Mutations | | | p-value |
| --- | --- | --- | --- | --- | --- |
|  |  | Susceptible | I54V, N88S | V82A, I84IV |  |
| ATV | High-Level Resistance | 0.0% | 1.8% | 1.8% | <.001 |
|  | Low-Level Resistance | 0.0% | 0.0% | 0.0% |  |
|  | Susceptible | 96.4% | 0.0% | 0.0% |  |
| DRV | High-Level Resistance | 0.0% | 0.0% | 0.0% | <.001 |
|  | Low-Level Resistance | 0.0% | 0.0% | 1.8% |  |
|  | Susceptible | 96.4% | 1.8% | 0.0% |  |
| LPV | High-Level Resistance | 0.0% | 0.0% | 1.8% | <.001 |
|  | Low-Level Resistance | 0.0% | 1.8% | 0.0% |  |
|  | Susceptible | 96.4% | 0.0% | 0.0% |  |

NRTI Resistance Mutations with Different Drugs

| Drug | Resistance | Mutations | | | | | | | p-value |
| --- | --- | --- | --- | --- | --- | --- | --- | --- | --- |
|  |  | D67N, M184V | M184 Deletion | M184V | M184V, T215SY | M41L | M41ML, L74LI, V75VI, M184V, T215TNS | Susceptible |  |
| 3TC | High-Level Resistance | 1.8% | 0.0% | 21.4% | 1.8% | 0.0% | 1.8% | 0.0% | <.001 |
|  | Low-Level Resistance | 0.0% | 0.0% | 0.0% | 0.0% | 0.0% | 0.0% | 0.0% |  |
|  | Susceptible | 0.0% | 1.8% | 0.0% | 0.0% | 1.8% | 0.0% | 69.6% |  |
| ABC | High-Level Resistance | 0.0% | 0.0% | 0.0% | 0.0% | 0.0% | 1.8% | 0.0% | <.001 |
|  | Low-Level Resistance | 1.8% | 0.0% | 21.4% | 1.8% | 0.0% | 0.0% | 0.0% |  |
|  | Susceptible | 0.0% | 1.8% | 0.0% | 0.0% | 1.8% | 0.0% | 69.6% |  |
| AZT | High-Level Resistance | 0.0% | 0.0% | 0.0% | 0.0% | 0.0% | 1.8% | 0.0% | <.001 |
|  | Intermediate Resistance | 0.0% | 0.0% | 0.0% | 1.8% | 0.0% | 0.0% | 0.0% |  |
|  | Low-Level Resistance | 0.0% | 0.0% | 0.0% | 0.0% | 1.8% | 0.0% | 0.0% |  |
|  | Susceptible | 1.8% | 1.8% | 21.4% | 0.0% | 0.0% | 0.0% | 69.6% |  |
| FTC | High-Level Resistance | 1.8% | 0.0% | 21.4% | 1.8% | 0.0% | 1.8% | 0.0% | <.001 |
|  | Low-Level Resistance | 0.0% | 0.0% | 0.0% | 0.0% | 0.0% | 0.0% | 0.0% |  |
|  | Susceptible | 0.0% | 1.8% | 0.0% | 0.0% | 1.8% | 0.0% | 69.6% |  |
| TDF | High-Level Resistance | 0.0% | 0.0% | 0.0% | 0.0% | 0.0% | 0.0% | 0.0% | <.001 |
|  | Low-Level Resistance | 0.0% | 0.0% | 0.0% | 0.0% | 0.0% | 1.8% | 0.0% |  |
|  | Susceptible | 1.8% | 1.8% | 21.4% | 1.8% | 1.8% | 0.0% | 69.6% |  |

NNRTI Resistance Mutations with Different Drugs

| Drug | Resistance | Mutations | | | | | | | | | | | | | | p-value |
| --- | --- | --- | --- | --- | --- | --- | --- | --- | --- | --- | --- | --- | --- | --- | --- | --- |
|  |  | E138A | K103N | V179DV | K103N, L100LI | K103N, V179T | K103N, P225H | K103N, H221Y, P225H | A98AG, V179VD, Y188L | K103N, P225H, K238T | K101E, K103N, G190A | K103N, Y188Del, M230L | K103KN, V106VI, V108VIM, E138EA | V106I | None |  |
| EFV | High-Level Resistance | 0.0% | 5.4% | 0.0% | 1.8% | 1.8% | 1.8% | 1.8% | 1.8% | 1.8% | 1.8% | 1.8% | 1.8% | 0.0% | 0.0% | <.001 |
|  | Potential Low-Level Resistance | 0.0% | 0.0% | 1.8% | 0.0% | 0.0% | 0.0% | 0.0% | 0.0% | 0.0% | 0.0% | 0.0% | 0.0% | 0.0% | 0.0% |  |
|  | Susceptible | 5.4% | 0.0% | 0.0% | 0.0% | 0.0% | 0.0% | 0.0% | 0.0% | 0.0% | 0.0% | 0.0% | 0.0% | 1.8% | 69.6% |  |
| ETR | Low-Level Resistance | 0.0% | 0.0% | 0.0% | 0.0% | 0.0% | 0.0% | 0.0% | 0.0% | 0.0% | 0.0% | 0.0% | 1.8% | 0.0% | 0.0% | <.001 |
|  | Intermediate Resistance | 0.0% | 0.0% | 0.0% | 1.8% | 0.0% | 0.0% | 0.0% | 1.8% | 0.0% | 1.8% | 1.8% | 0.0% | 0.0% | 0.0% |  |
|  | Susceptible | 0.0% | 5.4% | 0.0% | 0.0% | 1.8% | 1.8% | 0.0% | 0.0% | 1.8% | 0.0% | 0.0% | 0.0% | 0.0% | 69.6% |  |
|  | Potential Low-Level Resistance | 5.4% | 0.0% | 1.8% | 0.0% | 0.0% | 0.0% | 1.8% | 0.0% | 0.0% | 0.0% | 0.0% | 0.0% | 1.8% | 0.0% |  |
| NVP | High-Level Resistance | 0.0% | 5.4% | 0.0% | 1.8% | 1.8% | 1.8% | 1.8% | 1.8% | 1.8% | 1.8% | 1.8% | 1.8% | 0.0% | 0.0% | <.001 |
|  | Potential Low-Level Resistance | 0.0% | 0.0% | 1.8% | 0.0% | 0.0% | 0.0% | 0.0% | 0.0% | 0.0% | 0.0% | 0.0% | 0.0% | 1.8% | 0.0% |  |
|  | Susceptible | 5.4% | 0.0% | 0.0% | 0.0% | 0.0% | 0.0% | 0.0% | 0.0% | 0.0% | 0.0% | 0.0% | 0.0% | 0.0% | 69.6% |  |
| RPV | High-Level Resistance | 0.0% | 0.0.% | 0.0% | 1.8% | 0.0% | 0.0% | 0.0% | 1.8% | 0.0% | 1.8% | 1.8% | 0.0% | 0.0% | 0.0% | <.001 |
|  | Low-Level Resistance | 5.4% | 0.0% | 0.0% | 0.0% | 0.0% | 0.0% | 1.8% | 0.0% | 0.0% | 0.0% | 0.0% | 1.8% | 0.0% | 0.0% |  |
|  | Potential Low-Level Resistance | 0.0% | 0.0% | 1.8% | 0.0% | 0.0% | 0.0% | 0.0% | 0.0% | 0.0% | 0.0% | 0.0% | 0.0% | 1.8% | 0.0% |  |
|  | Susceptible | 0.0% | 5.4% | 0.0% | 0.0% | 1.8% | 1.8% | 0.0% | 0.0% | 1.8% | 0.0% | 0.0% | 0.0% | 0.0% | 69.6% |  |
